# The Immune Landscape of Papillary Thyroid Cancer in the Context of Autoimmune Thyroiditis

**DOI:** 10.3390/cancers14174287

**Published:** 2022-09-01

**Authors:** Fabiana Pani, Paola Caria, Yoshinori Yasuda, Miyara Makoto, Stefano Mariotti, Laurence Leenhardt, Solmaz Roshanmehr, Patrizio Caturegli, Camille Buffet

**Affiliations:** 1 Service des Pathologies Thyroïdiennes et Tumeurs Endocrines, AP-HP, Hôpital Pitié-Salpêtrière, Sorbonne Université, GRC n°16, GRC Tumeurs Thyroïdiennes, 75013 Paris, France; 2 Department of Biomedical Sciences, Biochemistry, Biology and Genetics Unit, University of Cagliari, Cittadella Universitaria di Monserrato, SP 8, Km 0.700, Monserrato, 09042 Cagliari, Italy; 3 Department of Endocrinology and Diabetes, Nagoya University Graduate School of Medicine, Nagoya 466-8550, Japan; 4 Inserm, Centre d’Immunologie et des Maladies Infectieuses-Paris (CIMI-PARIS), AP-HP Hôpital Pitié-Salpêtrière, Sorbonne Université, 75013 Paris, France; 5 Department of Medical Sciences and Public Health, Endocrinology Unit, University of Cagliari, Monserrato, 09042 Cagliari, Italy; 6 Division of Immunology, Department of Pathology, The Johns Hopkins School of Medicine, Baltimore, MD 21205, USA

**Keywords:** autoimmune thyroiditis, papillary thyroid cancer, immune check point inhibitors, tumor infiltrated-lymphocytes, tumor-infiltrated B lymphocytes

## Abstract

**Simple Summary:**

The association between papillary thyroid cancer and Hashimoto’s thyroiditis went through a long-standing human debate recently elucidated by the establishment of a novel mouse model. Papillary thyroid carcinoma is an excellent model for studying the tumor immune microenvironment because it is naturally accompanied by immune cells, making it a good candidate for the treatment with immune checkpoint inhibitors.

**Abstract:**

Papillary thyroid cancer (PTC) often co-occurs with Hashimoto’s thyroiditis, an association that has long been reported in clinical studies, remaining controversial. Experimental evidence has recently shown that pre-existing thyroiditis has a beneficial effect on PTC growth and progression by a distinctive expansion of effector memory CD8 T cells. Although the link between inflammation and PTC might involve different components of the immune system, a deep characterization of them which includes T cells, B cells and tertiary lymphoid structures, Mye-loid cells, Neutrophils, NK cells and dendritic cells will be desirable. The present review article considers the role of the adaptive and innate immune response surrounding PTC in the context of Hashimoto’s thyroiditis. This review will focus on the current knowledge by in vivo and in vitro studies specifically performed on animals’ models; thyroid cancer cells and human samples including (i) the dual role of tumor-infiltrating lymphocytes; (ii) the emerging role of B cells and tertiary lymphoid structures; (iii) the role of myeloid cells, dendritic cells, and natural killer cells; (iv) the current knowledge of the molecular biomarkers implicated in the complex link between thyroiditis and PTC and the potential implication of cancer immunotherapy in PTC patients in the context of thyroiditis.

## 1. Autoimmune Thyroiditis and Papillary Thyroid Cancer: New Insights

### 1.1. Human Data: A Long-Standing Debate

Thyroid cancer has the highest worldwide incidence and prevalence among all endocrine cancers [1,2], with papillary thyroid carcinoma (PTC) being the most frequent subtype [3]. Its incidence has markedly increased over the past several decades [2,4], in part due to the widespread use of diagnostic radiologic studies, which has led to the incidental discovery of small thyroid tumors of uncertain clinical significance [5,6]. Autoimmune thyroiditis, also known as Hashimoto thyroiditis, was first considered as a risk factor for PTC in 1955 [7], along with a family history of PTC [8], therapeutic and diagnostic irradiation [5], and inadequate dietary intake of iodine [9]. After the initial 1955 report [7], several studies have examined the PTC-thyroiditis association (summarized in Table 1), especially after the introduction of cancer immunotherapy to the clinical practice [10,11,12,13,14,15,16,17].

Scholars have long debated whether this association represents the coexistence of two independent but common diseases that are brought together by the increased use of thyroid ultrasound and fine-needle aspiration, or if it instead reflects a true cause–effect relationship [10,11,12,13,18,19,20,21,22,23,24,25,26,27,28]. Some studies have suggested that thyroiditis attenuates PTC severity [12,13,20,24,25,29,30,31], while others have concluded that thyroiditis promotes the progression of PTC [10,17,18,19,32]. Recently, McLeod et al. [32] conducted a case-control study nested within the United States Army personnel who served between 1996 and 2014. They reported that having thyroid antibodies 3 to 10 years before the clinical diagnosis of PTC was associated with an increased risk of PTC. Although the increase was in part mediated by thyroid autoimmunity awareness, this finding would suggest that thyroid autoimmunity (as judged by the presence of serum thyroid antibodies) has a detrimental effect on PTC. However, the same study also reported that, when thyroid autoimmunity had been diagnosed, PTC was smaller and less commonly metastasized to lymph nodes, thus suggesting a beneficial effect of thyroid autoimmunity on PTC. Overall, the true nature of the PTC-thyroiditis association remains difficult to dissect from human observational studies. In humans, the time of onset of the two diseases cannot be determined with certainty, and their diagnostic criteria greatly vary: while PTC diagnosis is based on a morphological analysis (either from cytopathology or surgical pathology specimens), the diagnosis of thyroiditis is based on the presence of thyroid antibodies in the peripheral blood and ultrasonographic characteristics, which do not recapitulate the complexity of the immune infiltrate present in the thyroid glands affected by Hashimoto thyroiditis.

For these reasons, animal models have been developed to gain insights into the complexity of the thyroid autoimmunity–PTC relationship. 

**Table 1:** Summary of surgical, cytological, and pathological human studies.

Scheme	Study Method (Thyroiditis Diagnosis)	State, Country	Sample Size (n. Patients)	Prevalence of PTC in Thyroiditis %	Reference
Dailey, 1955	Retrospective (histology)	USA	278	12.6	[7]
Okayasu, 1995	Retrospective (histology)	USA, JAPAN	1046	46.2–76.0	[29]
Buyukasik, 2011	Retrospective (histology)	TURKEY	917	19.5	[16]
Cipolla, 2005	Retrospective (serum; histology)	ITALY	225	27.6	[27]
Larson, 2007	Retrospective (histology)	USA	812	37.7	[21]
Bradly, 2009	Retrospective (histology)	USA	678	12.0	[22]
Siriweera, 2010	Retrospective (histology)	SRI LANKA	5357	9.46	[26]
Jancovic, 2013	Review	USA	9431 (8 studies)	27.56 (mean)	[23]
Chen, 2013	Retrospective (* NA)	TAIWAN	7605	NA	[18]
Castagna, 2014	Retrospective (Serum, FNAC)	ITALY	2054	4.5	[12]
Azizi, 2014	Prospective (serum, FNAB, Histology)	TURKEY	2023	11	[19]
Moon, 2018	Review	SOUTH KOREA	44,034 (71 studies)	39.2 (mean)	[28]
Radetti, 2019	Retrospective (serum, FNAB, Histology)	ITALY	904	5.7	[31]
Boi, 2018	Retrospective (FNAB, serum)	ITALY	484	31	[10]
Pilli, 2019	Retrospective (histology)	ITALY	375	20	[25]
Rotondi, 2021	Retrospective (histology)	ITALY	189	24.3	[24]
McLeod, 2022.	Case/control (Serum, histology)	USA	952 (451 cases and matched controls)	58	[32]

Abbreviation: * NA = not available.

### 1.2. Experimental Data: Animal Models

**Figure 1:**
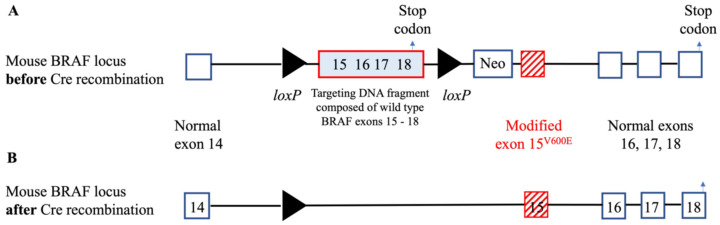
The BRAF knock-in mouse model: the *loxP* genomic structure. (**A**) In this model, the mutated version of BRAF exon 15, the one that contains the V600E mutation, was inserted into the mouse BRAF locus between exon 14 and exon 16. This mutated version of exon 15 was preceded by a floxed *loxP* DNA fragment containing the last four exons, 15, 16, 17, and 18, of mouse wild type BRAF. When CRE recombinase is not around, the mouse expresses the normal version of BRAF, ending transcription (see arrow). (**B**) When CRE is expressed instead, this section is cut out, and the mouse expresses the mutated version of exon 15, followed by the normal exons 16 to 18.

Animal models are a fundamental tool for cancer investigators and provide insights into tumor behavior that cannot be reproduced by an in vitro system [33]. Overall, three general approaches have been used to induce thyroid cancer in animals: administration of carcinogenic compounds, subcutaneous or intra-thyroidal implantation of tumor cells into immunocompromised mice or genetic manipulations [34,35,36]. Chemical compounds can be used to induce goiter, which is occasionally followed by the development of follicular neoplasms [37], but they have limited utility for pre-clinical studies. Implantation of patient-derived thyroid cancer lines into the thyroid of nude mice nicely recapitulates several features of human carcinogenesis [38,39,40], although these experiments are technically demanding and require dedicated surgical facilities. Genetic manipulations provide the most flexible and affordable to study thyroid carcinogenesis, and typically originate from pivotal observations made in patients. For example, the oncogenic mutation at codon 600 of the BRAF kinase (valine to glutamic acids) has been reported in PTC and several other human cancers. Dankort et al. created a targeting DNA construct composed of the normal BRAF exons 15–18, flanked by loxP sites and followed at the 3′ end by the mutated version of exon 15 (where codon 600 is located) (Figure 1A) [41]. These mice are phenotypically normal until crossed to mice that express the bacteriophage protein Cre under control of a promoter of choice, such as under the control of the thyroperoxidase (TPO) or thyroglobulin (Tg) promoter to direct Cre expression in thyroid cells [42,43,44]. In the crossed mice, Cre excises in the cells where it is expressed the fragment of genomic DNA flanked by the two loxP sites leaving, in the case of the BRAF construct, the normal BRAF exon 14 followed by the mutated exon 15, thus recreating the oncogenic version (V600E) of the BRAF kinase (Figure 1B). In this way, the investigator can spatially control the expression of the oncogenic BRAF in the body location of their choice (for example, in the thyroid gland when the Cre sequence is preceded by the TPO promoter). In addition, because the DNA sequence for Cre is fused with the DNA sequence encoding the portion of the human estrogen receptor that binds tamoxifen, the expression of the oncogenic BRAF can also be controlled temporally: that is, it will be induced only when mice are injected with tamoxifen. The TPO promoter-Cre-estrogen receptor mouse has been crossed to other strains as to mimic different aspects of carcinogenesis. For example, crossing it to mice carrying the BRAFV600E mutation and lacking the tumor suppressor gene p53 causes an aggressive form of PTC that progresses to anaplastic thyroid cancer [42]. Despite recent progress, few models with spontaneous mutations have been generated [45]. A list of the most relevant experimental models of thyroid oncogenesis using transgenic animals is summarized in Table 2.

**Figure 2:**
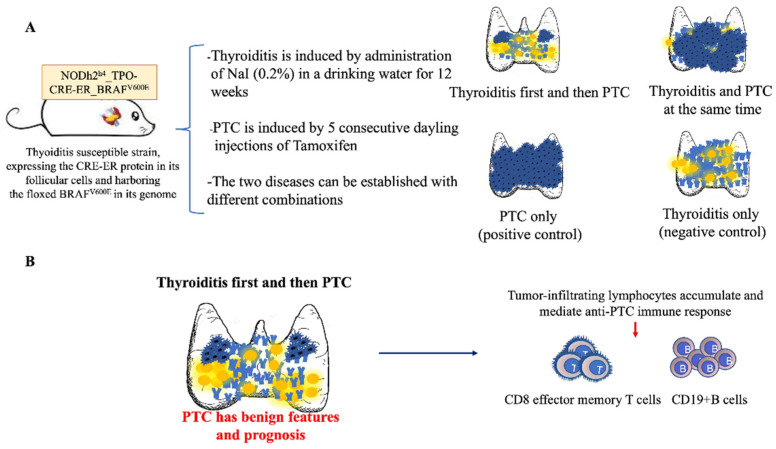
Mechanism underlying the beneficial role of pre-existing thyroiditis on PTC growth and progression in a double transgenic mouse model NOD.H2^h4^_TPO-CRE-ER_BRAF^v600E^. (**A**) autoimmune thyroiditis and PTC induction. Autoimmune thyroiditis was induced by administration of sodium iodide (NaI) in the drinking water for 12 consecutive weeks. PTC is induced by five consecutive daily injections of Tamoxifen (100 mg/Kg i.p.). The two diseases can then be established with various combinations: Thyroiditis first and then PTC; Thyroiditis and PTC induced at the same time; PTC only (positive control); Thyroiditis only (negative control). (**B**) Pre-existing iodine-induced thyroiditis as a primary event in PTC development has a beneficial effect leading to small PTC foci and higher lymphocytic infiltration characterized by CD8 effector memory T cells and CD19+ B cells.

Autoimmune thyroiditis has been modeled in animals since the mid 1950s [46,47,48]. It was originally induced by the injection of thyroid proteins or thyroglobulin mixed with a strong adjuvant. Then, in the early 1990s, Linda Wicker’s laboratory, while studying genetic loci that confer susceptibility to type 1 diabetes in the NOD mouse, serendipitously identified a congenic strain that lost the diabetes phenotype typical of the parental strain and instead acquired the development of thyroiditis at low incidence, an incidence that could be greatly enhanced by the administration of iodine in the drinking water [49]. This congenic strain, named NOD-H2^h4^, carries the thyroiditis susceptible k allele at the MHC class II A locus (K^k^, A^k^, E^0^, D^b^), and develops a chronic, lymphocyte rich form of thyroiditis [50] associated with the development of Tg antibodies (Abs) and then TPO Abs [51]. In addition to CD4+ and CD8+ T cells and B cells, the thyroid infiltrate also contains macrophages, natural killer cells, and dendritic cells [52,53]. We exploited the thyroiditis susceptibility of the NOD-H2^h4^ strain to create a new mouse model where autoimmune thyroiditis and PTC can be induced in a temporally definable fashion [54]: we backcrossed onto the NOD-H2^h4^ strain the TPO-CRE-ER transgenic strain [42] and the BRAF^V600E^ knock-in strain [41] for a minimum of 16 generations [54]. After these extensive backcrossing’s, TPO-CRE-ER transgenic and BRAFV600E knock-in mice were intercrossed for two generations to obtain mice that carry the BRAF^V600E^ mutation under control of the thyroidal Cre recombinase on a genetic background (that of the NOD.H2^h4^ strain_) that is susceptible to thyroiditis. This new strain, which we labeled NOD.H2^h4^_TPO-CRE-ER_BRAF^v600E^, develops PTC when injected with Tamoxifen and autoimmune thyroiditis when iodine is added to their drinking water (Figure 2A). Without Tamoxifen injections, PTC might be developed only in 20% of cases, likely a consequence of low-level constitutive expression of the transgenic BRAFV600E mutation [55]. We used this new mouse model to assess whether autoimmune thyroiditis has a different effect on PTC according to whether it develops concomitantly with PTC or if instead it precedes the development of PTC. We found that PTC is less common and less aggressive in mice that had first developed thyroiditis than in those where PTC and thyroiditis were induced at the same time. The thyroid glands of mice with pre-existing thyroiditis featured an abundant mononuclear cell infiltrate characterized by abundant effector memory CD8+ T cells and CD19+ B cells. These subsets of tumor infiltrating lymphocytes have been associated with favorable prognosis (Figure 2B). Effector memory CD8 T cells are in fact key mediators of the anti-tumor response: they expand rapidly upon antigen restimulation and secrete a large amount of pro-inflammatory cytokines [56]. Similarly, tumors containing B cells, especially when they compartmentalize into germinal centers, have a more benign phenotype, suggesting that B cells could be harnessed to develop novel forms of cancer immunotherapy [57]. Overall, findings from this mouse model suggest that there are different forms and gradations of autoimmune thyroiditis, and therefore different effects of PTC. A florid form of thyroiditis with prominent germinal centers may control and mitigate a PTC that later arises. On the contrary, scattered lymphocytes infiltrating the thyroid gland may not influence at all PTC oncogenesis, independently of whether they were present before the emergence of PTC or not.

**Table 2:** Experimental Models of thyroid oncogenesis in transgenic animals.

Mouse Line/Rat	Promoter/Expression	Original Strain	Pathology	Thyroid Function	References
RET/PTC1	Rat Tg, thyroid	C57BL/6J	4/18 (22%) diagnosed with * PTC starting at 8 months	NA	[37]
RET/PTC1	Bovine Tg, thyroid	FVB /N	100% mice with multifocal * PTC	Congenital hypothyroidism	[38]
RET/PTC3	Bovine Tg, thyroid	CH3/He	4/13 (31%) * PTC by 3 months and 6 /11 (55%) over 3 months	Normal	[39]
RET/PTC3	Bovine Tg, thyroid	FVB/N	7/12 (58%) * PTC-like neoplasia over 5 months	Primary hypothyroidism	[39,40]
TBP-3743	TPO, BRAF^V600E^/WT, Trp^53^	B6129SF1/J	100% mice with anaplastic thyroid cancer	More than ~1000-fold elevation of TSH	[41,42]
TPOCREER/BRAF^V600E^	TPO, BRAF^V600E^, Trp^53^	C57BL/6J	100% mice with multifocal * PTC 12 weeks post-TAM injection’s	Less than ~10-fold elevation of TSH	[45]
TPO KRAS^G12D^, PTEN^LL^	TPO	129Sv	100 % with follicular thyroid carcinoma	TSH autonomy (TSH drastically reduced with increased T4)	[47]
NODH2^H4^_TPO-CRE-ER_BRAF^V600E^	TPO, BRAF V600E under CRE expression	NODH2^H4^/C57BL/6J	Autoimmune thyroiditis and small/unifocal * PTC	Severe hypothyroidism in concomitant and no thyroiditis	[54]

Abbreviation. * PTC = Papillary thyroid carcinoma.

### 1.3. PTC and Thyroiditis: In Vitro Study

In vitro experimental models are the most used research tool for cancer studies [58]. In the past years, the two-dimensional (2D) monolayer culture has been widely used for studying different kinds of tumors but cannot accurately reflect the in vivo tissue pattern. Conversely, the 3D cultures have been gaining in popularity and are closely able to mimic in vivo conditions [59,60,61]. Although different works are available using PTC-derived cell lines for studying the thyroid tumorigenesis [62,63,64,65,66,67,68,69], very few in vitro studies have been performed to increase knowledge of the relationship between thyroiditis and PTC. Denning et al. [70] demonstrated that Nthy-ori cells (normal thyroid cell line) showed over-expression of immune related genes upon exposure of thyroid lymphocytes from autoimmune thyroiditis patients, suggesting a possible contribution of these genes to the pathogenesis of thyroiditis. In the same work, co-culturing the TPC-1 cell line (harboring *RET/PTC-1* rearrangement) with normal thyroidal lymphocytes, they can show that this cell line may dampen an immunogenic response in the thyroid, which could possibly facilitate PTC development. Moreover, using a rat thyroid cell line (PCCL3), it has been demonstrated that *RET/PTC3* can induce expression of NF-κB DNA-binding activity leading to increase proinflammatory cytokine secretion by thyroid cells [71]. Other genes involved in immune response and inflammation related to RET/PTC3 activation in thyroid PCCL3 cells are prostaglandin E2 (PGE2), microsomal prostaglandin E synthase1 (mPGES1), and cyclooxygenase2 (COX2) [72,73]. Recently, other groups evaluated the role of different interleukins in cells of PTC associated with thyroiditis [74,75] demonstrating that treatment of PTC-derived cell lines (TPC-1 and K1) promotes tumor antigenicity in PTC by expression of MHC class I possibly due to PD-1/PD-L1 pathway. Nevertheless, a fascinating work was published by Xiao and colleagues in 2021 [76]. Interestingly, using cells obtained from Hashimoto’s thyroiditis or PTC tissues, they were able to generate organoids derived from patients with PTC and, for the first time, from Hashimoto’s thyroiditis. Histological examination of Hashimoto’s organoids displayed a thyroiditis-like morphology enhancing expression of GAL3, a biomarker of PTC, which was maximally expressed in PTC derived organoids [76]. These results indicate that thyroidal organoids may be used as a new in vitro model to study the pathogenesis of both autoimmune thyroiditis and PTC and suggest that Hashimoto’s thyroiditis may share proliferative capacities with PTC, leading to tumoral progression in vivo. 

Taken together, the above experimental data support the notion that several phenomena linked to proliferative and inflammatory stimuli may contribute to explain the epidemiological association of PTC with autoimmune thyroiditis in humans. On the other hand, the complex interaction of the immune cells in PTC and in autoimmune thyroiditis also provides the basis to consider a role of PTC-specific protective immune response. In the next paragraph, we will focus on the role of the single components of the immune cells in Hashimoto’s thyroiditis and PTC human tissues, supporting evidence that PTC-immune response is tightly linked to a better clinical outcome. 

## 2. Adaptive and Innate Immune Response Surrounding PTC in the Context of Autoimmune Thyroiditis

### 2.1. T Lymphocytes and Tumor-Infiltrating Lymphocytes (TILs): General and New Concepts

The main pathologic feature of autoimmune thyroiditis is the presence of lymphocytic infiltration, primarily composed of T lymphocytes following follicular disruption, leading to a gradual atrophy and fibrosis of the thyroid gland [77].

T lymphocytes, distinguished into CD4+ and CD8+ T cells subsets, are part of the adaptive arm of the immune response and are necessary for the onset and progression of several autoimmune diseases [78,79,80], including thyroid autoimmunity [81]. Particularly, CD4+ T cells have been reported as the main players in the human chronic autoimmune thyroiditis [82].

Overall, T cell subsets were originally classified into naïve, effector, and memory cell populations [83]. Briefly, naïve T cells, which have not been exposed to antigens, possess strong proliferative potential after antigen stimulation. Memory T cells are typically differentiated into distinct subsets based on phenotypic definitions, and each subset has specific roles in immunity [56,84,85]. Central memory and effector memory T (EMT) cells circulate in the blood and target the secondary lymphoid tissues. EMT cells are the prime mediator of effector functions and strongly expand upon antigen restimulation secreting many inflammatory cytokines [86]. However, this subset has a limited power for population expansion and tends to become terminally differentiated, subsequently dying and became exhausted [87,88].

Recently, a new T-lymphocyte lineage called resident memory T (TRM) cells has been described to stay at local sites to respond immediately to secondary infection [89]. Moreover, this T-cell subset that is recognized by distinctive intra-cellular markers might also play a critical role in the tumor microenvironment [90] However, together with EMT, their role in autoimmune thyroiditis is not known. 

Another population, called T regulatory cells (Tregs), crucial for maintaining T-cell tolerance to self-antigens, is dysfunctional in autoimmune thyroiditis as recently demonstrated [91,92].

Chronic inflammation is currently regarded as an essential component of malignancies, including PTC [93], raising seminal questions about the immune system’s role in the pathogenesis of PTC [15,94]. 

It is known that the tumor microenvironment may profoundly influence the biological behavior of PTC [95]. Indeed, ultimately, the patient’s outcome can be positively or negatively influenced by the density and types of tumors-infiltrating lymphocytes (TILs) [96,97]. Different types of cancers, called “hot” such as metastatic melanoma [98], ovarian [99], rectal [100], and breast cancers [101] show a good outcome in the presence of TILs.

Several authors have demonstrated that, in human PTC, the density of lymphocytes is positively correlated with lower recurrence rate and improved overall survival [97,102], supporting the knowledge that T lymphocytes are more common in less aggressive forms of PTC [103,104,105,106].

T cell populations in PTC are largely expressed, including CD4+ helper T cells, classically known to direct the immune response through the production of cytokines and mediate protective host immune responses [103]. CD8+ T cells, together with EMT cells, can direct cytotoxicity to tumors, and are commonly found within, and surrounding thyroid tumors [86,103,107,108]. These effector TILs have been extensively characterized in the thyroid gland [102,109,110,111] and are often classified into functional subsets. 

An initial comprehensive analysis of CD8+ TRM cells, as another subset of TILs, in PTC samples, revealed a lower proportion of this population in the more advanced stage of disease, consistent with an anti-tumor phenotype [112,113], but further work is required to better understand their prognostic value. 

Contrary to helper T cells, Tregs switch off immune responses and have been identified as a key component of the CD4+ infiltrate in PTC [114]. These cells have been associated with increased frequency of lymph node metastases and recurrent disease rate [106,115]. 

### 2.2. B Lymphocytes and Tumor-Infiltrated B Cells: A Novel and Emerging Role in Anti-Tumor Immunity

Autoimmune thyroiditis is also characterized by the infiltration of B lymphocytes [116] that contributes to the immune response through the production of antibodies, antigen presentation to T cells, and the production of immune-modulating cytokines [117,118]. 

Overall, B lymphocytes possess distinct functions in both adaptive and innate humoral immune responses, and they are classified in different distinct subsets defined by the recognition of the CD19+ marker [119]. They have commonly been identified in primary PTC and accumulated within follicular structures in peritumoral regions [103,107,108,120]. In these organized follicles, they are probably capable of presenting tumor-antigen to tumor-specific T cells. Although no correlation has been found between the frequency of B cells and PTC severity, a deep characterization of tumor-infiltrating B cells (TIBs) might recognize a distinct role in the tumor eradication or promotion [108].

Accumulating experience in several solid tumors, such as head and neck cancers and glioblastoma, has suggested a correlation of TIBs and a good prognosis [121,122,123]. PTC immune cell infiltration is remarkably heterogeneous, and the role of this component in PTC development and progression is not fully explored. 

Yang et al. [124] compared the infiltration status in patients with or without lymph node metastases providing evidence that TIBs are positively correlated with TILs’ infiltration, and indicating a protective role of these immune cells in PTC. 

Interestingly, Pan and colleagues [125] recently investigated by single-cell RNA sequencing an enrichment pattern of TIBs in PTC tumor tissues that were concurrent with thyroiditis, but not in the PTC alone/without thyroiditis. They confirmed the indolent property of PTC when it is associated with a pre-existing thyroiditis explained by the expansion of this distinct signature of B cells [54].

Finally, the intrathyroidal infiltration is characterized not only by distinct B cells lymphocytes, but also by the development of follicles resembling secondary lymphoid structures that are defined as tertiary lymphoid structures (TLS) [92]. A deep characterization of a novel B cell subset, called B cells memory targeting CD20 + CD22 + markers, has been reported by [126]. Wu et al. found that the TLS are mainly infiltrated by this specific B cell subset able to adopt a specialized memory phenotype enhancing anti-tumor immunity. 

Taken together, these novel human data are too preliminary to support the potentially positive role of B lymphocytes and TIBs in PTC outcomes. Further experimental studies using our pre-clinical mouse model are necessary to gain insights on the mechanism(s) underlying the functional and biological role of these emerging fields. 

### 2.3. Mast Cells, Natural Killer Cells, Macrophages, Dendritic Cells, Myeloid Cells, and Neutrophils: The Innate Immune Response

Mast cells, a type of proinflammatory granulocyte, have been found at increased levels in PTC when compared to normal thyroid tissues, also associated with extra-thyroidal extension [114]. When activated, these cells can mediate thyroid cell dedifferentiation and invasion as described in both preclinical and in vitro studies [127,128]. 

Natural killer cells are known to play an important role in anti-viral and anti-tumor immunity [107]. Their role is controversial in PTC outcomes [107]. Some authors found an increased population in the low stage of disease compared to advanced stage [129], but others found the opposite [130]. 

Macrophages have been clearly identified in primary thyroid tumors expressing co-stimulatory molecules, known to be essential for the T-cell activation, more likely correlated with decreased survival [131,132]. However, pre-clinical studies using a mouse model with thyroiditis, and PTC showed clear but sparse infiltrating macrophages cells in the thyroid affected by PTC [54].

Dendritic cells are inefficient and commonly immature in PTC peritumoral tissues, and they fail to become mature in the established tumor [133]. 

Data about the role of myeloid cells in PTC and thyroiditis [130], broadly known as myeloid (granulocytic or monocytic) precursors that suppress the normal immune response, are very limited. 

Regarding the role of neutrophils, they are known to be involved in the acute phase of the inflammatory response [102,134] representing the first line of defense against extracellular microbes [135]. The density of tumor-associated neutrophils in colorectal cancers correlated with a better patient’s clinical outcome [136], although their functional roles are still controversial [137,138]. 

A new routinely available marker of the systemic inflammatory response is the neutrophil lymphocytic ratio (NLR), calculated by dividing the number of neutrophils by the number of lymphocytes usually from a peripheral blood sample. This prognostic marker is broadly applied in the oncologic field including also PTC [139,140,141]; when is increased (>3 as considered the normal value), it is correlated with a larger tumor volume and a higher risk of recurrence [140]. Using a cohort of 151 PTC patients, Lee et al. [141] studied the effect of baseline performance status and NLR on the progression-free survival (PFS), overall survival (OS), and objective response rate (ORR). They reported a significant decrease in NLR in low-risk PTC patients, although a significantly increased value was observed in patients with an incomplete structural response.

On the contrary, among patient’s refractory to iodine, thus considered at high risk, associated with a baseline NLR < 3, they observed a trend toward an increase of ORR compared with patients with NLR > 3 [139]. 

It would be interesting to investigate the role of this new inflammatory marker also in PTC patients with or without pre-existing thyroiditis to investigate if there is a positive correlation in the PTC outcome. Further studies will be necessary to add it as a new prognostic biomarker in the complex link between thyroiditis and PTC.

**Figure 3:**
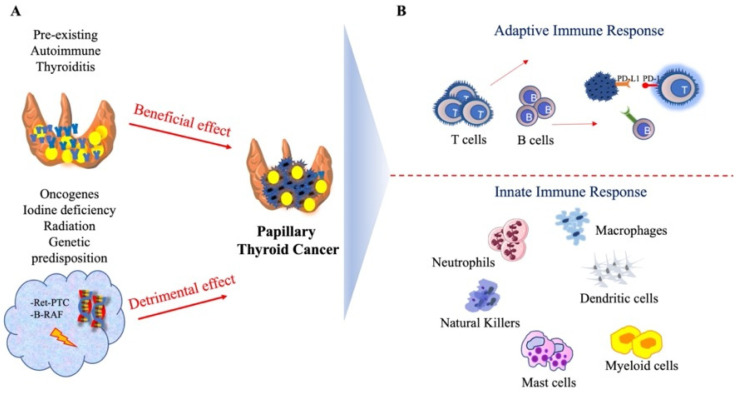
The complex association between autoimmune thyroiditis and papillary thyroid cancer (PTC). (**A**) autoimmune thyroiditis and its beneficial effect in PTC development; oncogenes, radiation, genetic predisposition and iodine deficiency and their detrimental effect in PTC development; (**B**) different components of the adaptive and innate immune response surrounding PTC. PTC = Papillary thyroid carcinoma.

A global representation of T cell subsets in autoimmune thyroiditis and PTC, as well as the schematic representation of the complex association between thyroid (auto)immunity and PTC, are displayed in Figure 3.

### 2.4. Single-Cell Transcriptome Signature of PTC and Autoimmune Thyroiditis

PTC is a highly heterogeneous tumor with different cell populations including lymphocytes and myeloid cells that influence tumor initiation, progression, and treatment resistance [142]. 

Single-cell RNA sequencing (scRNA-seq) is a useful approach to better understand the interaction between malignant cells and the surrounding immune cells used in different cancers, including also papillary thyroid carcinomas [143,144,145]. A recent scRNA-seq study demonstrated a different molecular PTC phenotype according to the tumor microenvironment (TME) and gender revealing differences in cell differentiation and immune microenvironment of papillary thyroid carcinoma [145]. Another most recent study defined the molecular evolutionary pathway from PTC to anaplastic thyroid cancer at single-cell resolution using 46,205 cells [146]. Collectively, their findings provided insights into the heterogeneity and molecular evolution of thyroid cancer. 

More importantly, Pan et al. [125] constructed the single-cell transcriptome landscape of PTC and delineated, for the first time, a distinct transcriptional state of PTC with and without thyroiditis. Authors demonstrated that the presence of BRAFV600E positive tumor cells and high expression of different genes (such as TACSTD2, CLDN3, CTSC, and B2M) correlate with disease-free survival and metastatic lymph nodes. Samples from PTC patients without thyroiditis showed high expression of TFF3 while PTC with thyroiditis high expression of CCDC80. It has been shown that this protein played a role as a putative tumor suppressor gene [147] and could positively regulate E-cadherin expression preventing cancer progression. Furthermore, different ligand–receptor pairs between non-immune cells, infiltrating myeloid cells, and lymphocytes were detected. 

These findings provide a deeper insight into the cellular interactions that might prompt the pathogenesis of thyroiditis. 

### 2.5. Antigen Specificity in Autoimmune Thyroiditis and Correlation with PTC Outcome

The diagnosis of autoimmune thyroiditis relies on the demonstration of circulating antibodies to thyroid antigens (mainly TPO and Tg) that are characterized by epitope-specific cellular immunity of CD8+ lymphocytes [116,148]. Growing evidence is showing that PTC is surrounded by an abundant lymphocytic infiltration into the tumor site affecting clinical outcome [29,94,102,103,105,109,111,120]. The prognostic significance of the serum TPO and Tg antibodies in PTC patients with and without thyroiditis has been extensively evaluated [149,150,151,152,153,154] supporting the notion that results from PTC-patients associated with thyroiditis are more concordant than PTC without because their epitope pattern is more restricted. 

Ehlers et al. [154] investigated the prevalence of tumor epitope-specific T cells using a cohort of 211 patients (150 with PTC, 40 with autoimmune thyroiditis and 21 healthy controls). They found a significant higher frequency of TPO- and Tg-specific CD8+ T cells in PTC-thyroiditis patients compared with healthy controls. To evaluate the clinical impact of their findings, they investigated the HLA class-II phenotype revealing a distinct pattern of the HLA-DQ B1*03 responsible for an increased responsiveness of tumor epitopes in vitro able to suppress PTC tumor spread. 

In a very original study, using a cohort of Sardinian patients, Latrofa et al. [151] demonstrated that serum antibodies directed against thyroglobulin in patients with PTC are considered either a reflection of co-existing autoimmune thyroiditis or the response of the immune system to newly emerged tumor associated epitopes. In line with these data, in our pre-clinical mouse model [54], we observed an initial peak of thyroglobulin antibodies that might reflect the response to dominant and/or cryptic iodinated epitopes [51]. The long-term persistence of thyroglobulin antibodies in tamoxifen-treated mice, however, potentially suggests a true association of these antibodies with PTC development, resulting from the appearance of novel, tumor-related epitopes within the large thyroglobulin molecule. Further studies will be needed to support the evidence of epitope specific anti-tumor immunity in PTC patients.

## 3. Molecular Biomarkers of the Pathogenic Link between Thyroiditis and PTC

The molecular landscape of PTC is characterized by activating mutations in oncogenes, *BRAFV600E* being the most frequent (60%) followed by mutations in the different isoforms of RAS (8% of PTC), as well as gene fusions, such as RET/PTC [155]. Although the association between thyroiditis with PTC has been well-established [7,10,11,13,18,19,24,25,28,29], the definitive molecular link is still elusive. 

*RET/PTC* plays a role as a potential pathogenic link between thyroiditis and PTC. The first RET/PTC fusion was described by Wirtschafter and collaborators in 1997 [156]. In this paper, using a sensitive and specific reverse transcriptase-polymerase chain reaction (RT-PCR) assay, the authors were unable to find if chronic inflammation might facilitate the rearrangement, or conversely, RET/PTC rearrangement might promote chronic inflammation [94]. Since then, many studies, using in vitro strategy, have been carried out supporting this association [59,60,61,62,64,157]. Indeed, the relevance of *RET/PTC* in the association between thyroiditis and PTC is also underscored by a retrospective pathological study [158,159,160]. On the other hand, other groups have not found *RET/PTC* rearrangements in PTC-thyroiditis tissues [161,162].

Moreover, a recent meta-analysis [163] showed that *BRAFV600E* mutations seem to be less common in PTC patients with thyroiditis than in PTC patients without. Lim et al. [164] investigated the predictive role of *BRAF* mutation in 3130 PTC patients with thyroiditis. Interestingly, they found that coexistent autoimmune lymphocytic thyroiditis was significantly less prevalent in the *BRAF* mutant group [164]. Similar results were then obtained by other researchers [165], confirming these preliminary data. Additionally, it has been reported that there is a better clinical outcome when thyroiditis and *BRAFV600E* coexist together in patients with advanced stage PTC [166,167,168], suggesting a protective role of thyroiditis in PTC [169,170].

Other molecular biomarkers have also been investigated, using human tissues. Unger et al. [171] revealed a higher expression of the p53 homolog p63 protein in thyroiditis-PTC tissue when compared to normal thyroid tissue, indicating a potential role of p63 in the association between thyroiditis and PTC. Nevertheless, further studies are needed to confirm the role of p63 in the pathogenic link between thyroiditis and PTC. 

Currently, molecular inhibitors that block RET/PTC or BRAF kinase activity have shown substantial therapeutic effects in the experimental systems [172,173,174] and are currently being tested in several clinical trials [174]. Still, in some cases, they lost efficacy [175]. Other prognostic biomarkers using the immune system as a weapon are needed to explain why patients’ response is highly variable. 

## 4. Thyroiditis and PTC: Implications for Cancer Immunotherapy

Cancer immunotherapy has dramatically changed the approach to cancer treatment [176].

The aim of targeting the immune system to recognize and destroy cancer cells has been afforded to many patients; however, many challenges remain for achieving the goal of effective immunotherapy for all cancer patients. The recent success of the use of the checkpoint inhibitors (ICIs) ipilimumab (anti-CTLA4 antibody), pembrolizumab and nivolumab (anti-PD-1 antibody) have reinvigorated this field [177,178,179]. Focusing on their role, these antibodies block ligation of the CTLA-4 and PD-1 inhibitory receptors, potentially enhancing the patient’s existing antitumor T-cell response by acting directly on the effector T cells or inhibiting the suppressive effects of regulatory T cells [177,180]. These drugs, either alone or in combination, were recently approved by the US Food and Drug Administration in numerous solid tumors [181], but not in PTC yet. 

Some in vivo and in vitro studies [39,40,106,108,182,183] have demonstrated that ICIs could eliminate thyroid cancer cells, but it is unclear which ICIs might be the most effective. 

Several emerging ongoing clinical trials are recently now considering PTC patients, but the biological basis of the ICIs response to these patients is unknown [184,185]. A different hypothesis has been made by [186,187,188], all related to the modulation of the host immune system at the peritumoral or systemic level. Data about the correlation between the immune landscape of PTC and the possible implications for ICIs have been recently reported [14,189]. Interestingly, Sun and colleagues [14], using a transcriptomic analysis by a series of bioinformatic and machine learning approaches, were able to identify a specific immune-related genes (IRGs) and distinct immune clusters between PTC and normal thyroid tissues aiming at quantifying in each sample the “innate” and the “adaptive” immune response. They first demonstrated that PTC and normal thyroid tissue samples were clearly separated by a distinct pattern of IRGs. Among them, they isolated a PTC-cluster that clearly showed that a better ICI response signature, based on the T-infiltration score, mainly represented by a significant higher number of CD4+ and Treg cells.

In light of these above findings that immune infiltration might be correlated with PTC outcome, the need to further characterize the use of ICI drugs based on the presence of the onset of thyroiditis in PTC-patients is desirable. 

## 5. Conclusions

There is overwhelming evidence that autoimmune thyroiditis affects PTC’s natural history [7,11,15,102,169]. Despite having been reported in numerous clinical studies, the concept of the significant association between autoimmune thyroiditis and PTC remains controversial [10,24,102]. A new model to elucidate this long-standing debate has been recently generated using a genetic approach to induce both thyroiditis and PTC [54]. This pre-clinical study originally showed a distinctive feature of thyroid TILs from mice with pre-existing thyroiditis with an expansion of effector memory CD8 T cells, thus proposing it as an important event that halts or delays the onset and progression of PTC. The increase in thyroidal B cells associated with pre-existing thyroiditis could therefore also be an important event contributing to immune-mediated control of PTC. Moreover, the emerging role of tumor-infiltrated B cells, using human samples, showed an enrichment pattern of these subsets only in PTC tissues concurrent with thyroiditis but not in the PTC alone, suggesting a promising beneficial role of these subsets in the PTC behavior [125]. 

Finally, this pre-clinical mouse model could be a further step towards a better understanding of the complex relationship between autoimmunity, inflammation and thyroid cancer helping researchers to give some pathogenetic definitive answers. However, further translational studies will be necessary to validate and extend these results. Preliminary studies, using single-cell RNA sequencing analysis of peritumoral and intra-tumoral immune cells in PTC-tissue that are coexistent with thyroiditis, could be of paramount importance to elucidate their functions. Expanding knowledge in this field using human thyroid tissues with thyroiditis will successfully classify patient’s outcomes based on their immune profile.
